# Physiological Incest

**Published:** 1860-05

**Authors:** J. J. M. Angrar

**Affiliations:** Racine, Wis.


### PHYSIOLOGICAL INCEST.

Messrs. Editors of the Chicago *Medical Journal*:—In the April number of your Journal, I find an article, written by W. Boyd Powell, M. D., entitled, "Physiological Incest."

The cause of insanity, is a subject that deserves, yes, *demands*, the attention of the profession, in an *economical*, as well as *scientific*, point of view.

The Dr. thinks he has found the cause of "premature death and decay," as well as insanityand idiotcy—also can prognosticate the fate of the offspring, arising from the union of the temperaments. (?)

On account of the importance of the subject, and my interest in the same, please allow me to ask the Dr. a few questions, through the medium of your Journal:

1st. Are we always sure to have an amalgamation of the progenitor's temperaments, in the progeny?

2d. Are there any temperaments of such a nature, that if they should come together in progenitors, they would be neutralized in their progeny?

3d. What constitutes *compatibility* and *incompatibility* in the temperaments?

4th. Why will the progeny of the progenitors, whose temperaments are "3 and 4," be dead born?

5th. How do we *know* that the progeny springing from progenitors of certain temperaments, will die of tubercular disease—others become insane early in life, and others be born idiots?

6th. What does this mean?—"Two-thirds of the progenitors, indicated by 13, will be dead born, and the other third will die during infancy." If the two-thirds *die before they are born*, how can they ever become *progenitors*? And if the other third die before puberty, how can they ever become progenitors?

Respectfully,

J. J. M. Angrar, M. D.

Racine, April 15th, 1860.

